# Frequency of skin diseases in renal transplant recipients and patients with chronic kidney disease in a tertiary center: a cross-sectional study

**DOI:** 10.1590/1516-3180.2023.0148.R1.29112023

**Published:** 2024-02-23

**Authors:** Érica Cristina Vieira, Milena Soriano Marcolino, Antônio Carlos Martins Guedes, Mônica Maria Moreira Delgado Maciel, Wandilza Fátima dos Santos, Luciana Consoli Fernandes Pimentel, Paulo Rodrigues Gomes, Anita Bressan, Kátia de Paula Farah, Marcelo Grossi Araújo

**Affiliations:** I MD, Masters Student. Dermatologist, Attending physician, Dermatology Outpatient Clinic, Hospital das Clínicas, Universidade Federal de Minas Gerais / Empresa Brasileira de Serviços Hospitalares (UFMG/EBSERH) Belo Horizonte (MG), Brazil.; II MD, PhD. Associate Professor, Department of Internal Medicine, Medical School, Universidade Federal de Minas Gerais (UFMG), Belo Horizonte (MG), Brazil; Coordinator of Scientific Division, Telehealth Network of Minas Gerais, Hospital das Clínicas, Universidade Federal de Minas Gerais (UFMG), Belo Horizonte (MG), Brazil.; III MD, PhD. Dermatologist. Attending Physician, Dermatology Outpatient Clinic Hospital das Clínicas, Universidade Federal de Minas Gerais (UFMG), Belo Horizonte (MG), Brazil.; IV MD. Nephrologist, Attending physician. Instituto Mineiro de Nefrologia, Belo Horizonte (MG), Brazil.; V MD. Dermatologist, Attending physician, Dermatology Outpatient Clinic, Hospital das Clínicas, Universidade Federal de Minas Gerais (UFMG), Belo Horizonte (MG), Brazil.; VI MD. Dermatologist, Attending physician, Dermatology Outpatient Clinic, Hospital das Clínicas, Universidade Federal de Minas Gerais (UFMG), Belo Horizonte (MG), Brazil.; VII Masters Student. Analyst Programmer, Telehealth Network of Minas Gerais, Hospital das Clínicas, Universidade Federal de Minas Gerais (UFMG), Belo Horizonte (MG), Brazil.; VIII Medical Student, Medical School, Universidade Federal de Minas Gerais (UFMG), Belo Horizonte (MG), Brazil.; IX PhD. Associate Professor Department of Internal Medicine, Medical School, Universidade Federal de Minas Gerais (UFMG), Belo Horizonte (MG), Brazil Nephrology Outpatient Clinic, Hospital das Clínicas, Universidade Federal de Minas Gerais (UFMG), Belo Horizonte (MG), Brazil.; X PhD. Associate Professor, Medical School, Universidade Federal de Minas Gerais (UFMG), Belo Horizonte (MG), Brazil; Leprosy Clinic Coordinator. Dermatology Outpatient Clinic, Hospital das Clínicas, Universidade Federal de Minas Gerais (UFMG), Belo Horizonte (MG), Brazil.

**Keywords:** Skin diseases, Kidney transplantation, Chronic renal insufficiency, Immunosuppressive agents, Basal cell carcinoma, Squamous cell carcinoma, Dermatosis, Non-melanoma skin cancer, Renal transplant, Immunosupression, Kidney failure

## Abstract

**BACKGROUND:**

The prevalence of chronic kidney disease (CKD) has increased in the recent decades, along with the number of patients in the terminal stages of this disease, requiring transplantation. Some skin disorders are more frequent in patients with CKD and in renal transplant recipients (RTR).

**OBJECTIVES:**

To evaluate the frequency of skin diseases in RTR and patients with CKD receiving conservative treatment.

**DESIGN AND SETTING:**

This observational cross-sectional study recruited consecutive patients with CKD and RTR from a nephrology clinic at a teaching hospital in Brazil between 2015 and 2020.

**METHODS:**

Quantitative, descriptive, and analytical approaches were used. The sample was selected based on convenience sampling. Data were collected from dermatological visits and participants’ medical records.

**RESULTS:**

Overall, 308 participants were included: 206 RTR (66.9%, median age: 48 years, interquartile range [IQR] 38.0–56.0, 63.6% men) and 102 patients with CKD (33.1%, median age: 61.0 years, IQR 50.0–71.2, 48% men). The frequency of infectious skin diseases (39.3% vs. 21.6% P = 0.002) were higher in RTR than in patients with CKD. Neoplastic skin lesions were present in nine (4.4%) RTR and in only one (1.0%) patient with CKD. Among the RTR, the ratio of basal cell carcinoma to squamous cell carcinoma was 2:1.

**CONCLUSIONS:**

This study revealed that an increased frequency of infectious skin diseases may be expected in patients who have undergone kidney transplantation. Among skin cancers, BCC is more frequently observed in RTR, especially in those using azathioprine.

## INTRODUCTION

Chronic kidney disease (CKD) is a significant global public health problem with a major socioeconomic impact.^1,2^ Its worldwide prevalence is estimated at 10–16%;^1,3,4^ additionally, its prevalence has increased in the recent decades, along with the number of patients with terminal CKD requiring transplantation,^3,6^ mostly due to the increase in the prevalence of hypertension and diabetes mellitus.^4,5^

Patients with CKD are prone to skin abnormalities.^7,8^ These manifestations are often associated with impaired renal function and are more prevalent in end-stage disease,^7,8^ when the kidneys are unable to maintain appropriate levels of metabolic products, such as urea, creatinine, sodium, calcium, and phosphate, causing damage to several organs, including the skin.^9^

Kidney transplantation is the best treatment for patients with end-stage CKD;^3,10^ however, the immunosuppression required to maintain the graft can lead to various side effects and a greater susceptibility to infectious and neoplastic diseases.^11^ Besides immunosuppression itself, the mechanisms of action of immunosuppressive drugs and viral infections (oncogenic viruses) are associated with cutaneous disorders in renal transplant recipients (RTR).^12,13^

There is evidence that dermatological diseases affect the quality of life of patients with CKD^14^ and individuals who have undergone kidney transplantation.^15^ Several transplant centers do not have a dermatologist working with the transplant team, and dermatological abnormalities are often underdiagnosed and undertreated.^15^ Therefore, further research on the prevalence and presentation of skin diseases in solid organ transplant recipients and patients with CKD is essential. The prevalence and presentation of skin diseases is likely to vary in different regions of the world according to patient genetics, skin phototype, hygiene habits, sun exposure, immunosuppressive medications used, climate, and the prevalence of infectious agents.

## OBJECTIVE

This study aimed to evaluate the prevalence of dermatological abnormalities in patients with CKD receiving conservative treatment and in RTR treated at a tertiary academic center in southeastern Brazil.

## METHODS

### Data source and study participants

For this observational and cross-sectional study, consecutive patients treated between 2015 and 2020 were recruited from a reference center for nephrology and kidney transplantation at a Brazilian academic hospital. The sample was obtained through convenience sampling by inviting consecutive patients who were treated at the nephrology and kidney transplantation outpatient clinics of the hospital.

The eligibility criteria were: RTR regardless of the time elapsed since transplantation, patients diagnosed with CKD (defined as individuals with glomerular filtration rate [GFR] < 60 mL/min/1.73 m^2^ for at least 3 months) receiving conservative treatment, or patients with GFR ≥ 60 mL/min/1.73 m^2^ associated with markers of kidney damage or structural abnormalities detected by imaging; only patients ≥18 years of age were considered.^16^ Patients living with HIV and patients with CKD taking prednisone at ≥5 mg/day or taking other immunosuppressants were excluded from the study. The equation developed by the Chronic Kidney Disease Epidemiology Collaboration group (CKD-EPI) was used to calculate the GFR.^17^ CKD was classified into five stages according to the Kidney Disease Outcomes Quality Initiative of the National Kidney Foundation (KDOQI/NKF) classification.^18^

All patients underwent standard screening according to a previously established protocol to provide a solid, standardized assessment that included a dermatological perspective and variables of interest for kidney transplant/disease. Evaluation, diagnosis, treatment of skin diseases, biopsies, and direct mycological examinations were performed by attending dermatology physicians at the teaching hospital.

### Outcome measurements and group and subgroup analysis

The following independent variables were collected: age, sex, Fitzpatrick skin phototype, eye color, alcohol and tobacco use, presence of CKD, history of kidney transplantation, kidney function (estimated from the creatinine level or proteinuria), underlying disease that led to kidney transplantation, underlying disease that caused CKD, comorbidities, personal or family history of skin cancer, regular use of sunscreen, and previous sun exposure at work. The level of sun exposure considered was the highest exposure during the workday. Sunscreen application at least once per day was considered regular. Immunosuppressants were collected from in the RTR groups’ medical records.

The outcomes of interest were dermatological complaints and diagnoses of cutaneous disease. The skin diseases were divided into four groups: benign non-infectious, infectious (viral, bacterial, or fungal), preneoplastic, and neoplastic.

### Statistical analysis

Categorical variables were expressed as numbers and proportions, and continuous variables were expressed as medians and interquartile ranges. As we expected, there was an age difference between groups (RTR and CKD) and the prevalence of skin manifestations was affected by age; thus, the patients were stratified by age into three categories: 18–39 years, 40–59 years, and 60+ years. The Pearson’s chi-square test or the Fisher’s exact test was performed to determine the association between qualitative variables, and age groups/subgroups were compared using the Mann–Whitney U test. The statistical significance was set at P < 0.05. All statistical analyses were performed using the Statistical Package for the Social Sciences software (SPSS) v. 18.0 software for Windows (IBM, Armonk, NY, USA).

### Ethics

The study protocol was approved by the Universidade Federal de Minas Gerais Ethics Review Board (CAAE process number 38071114.8.0000.5149) on December 9^th^, 2014. Written informed consent was obtained from all participants.

## RESULTS

### Patient characteristics

Overall, 308 participants met the inclusion criteria for the study: 206 (66.9%) RTR and 102 (33.1%) patients with CKD receiving conservative treatment. The median age of the participants in the RTR group was 48.0 years (interquartile range [IQR]: 38.0–56.0), and 63.6% were men. When this group was stratified by age, most participants were in the subgroup of age 40–59 years (54.9%). The median age of the CKD group was 61.0 years (IQR: 50.0–71.0) and 48.0% were men (Table 1). Most patients in this group (54.9%) were 60+ years old. The demographic and clinical characteristics of both the groups are presented in Table 1.

**Table 1.** Demographics and clinical characteristics of renal transplant recipients and chronic kidney disease patients (n = 308)

	RTR n = 206	CKD n = 02	P
**Age (years)**	48.0 (38.0–56.0)	61.0 (50.0–71.0)	< 0.001^c^
18–39	59 (28.6%)	11 (10.8%)	
40–59	113 (54.9%)	35 (34.3%)	
60+	34 (16.5%)	56 (54.9%)	
Men	131 (63.6%)	49 (48.0%)	
**Kidney disease etiology**			< 0.001^b^
Hypertensive nephropathy	13 (6.3%)	7 (6.9%)	
Diabetes	14 (6.8%)	26 (25.5%)	
Glomerulopathy	55 (26.7%)	24 (23.5%)	
Polycystic kidney disease	10 (4.9%)	3 (2.9%)	
Genetic disease	9 (4.4%)	0	
Unknown	79 (38.3%)	32 (31.4%)	
Other	26 (12.6%)	10 (9.8%)	
**Comorbidities**			
Diabetes			< 0.001^b^
Type 1 diabetes	12 (5.8%)	3 (2.9%)	
Type 2 diabetes	11 (5.3%)	22 (21.6%)	
Post-transplant diabetes	35 (17.0%)	0	
Hypertension	122 (59.2%)	74 (72.5%)	0.024^a^
CAD	7 (3.4%)	12 (11.8%)	0.10^a^
Heart failure	3 (1.5%)	5 (4.9%)	0.121^a^
Obesity	5 (2.4%)	9 (8.8%)	0.018^a^
Hyperuricemia	25 (12.1%)	5 (4.9%)	0.064^a^
CKD stages*			0.001^b^
1	31 (15.0%)	9 (8.8%)	
2	87 (42.2%)	11 (10.8%)	
3A	32 (15.5%)	26 (25.5%)	
3B	30 (14.6%)	33 (32.4%)	
4	20 (9.7%)	21 (20.6%)	
5	6 (2.9%)	2 (2.0%)	
GFR**	64.2 (43.9–82.3)	44.1 (31.2–56.3)	0.000^a^
**Skin phototype**			0.286^b^
I	4 (1.9%)	0	
II	25 (12.1%)	6 (5.9%)	
III	86 (41.7%)	44 (43.1%)	
IV	53 (25.7%)	33 (32.4%)	
V	29 (14.1%)	16 (15.7%)	
VI	9 (4.4%)	3 (2.9%)	
**Tobacco use**	16 (7.8%)	20 (19.6%)	0.004^a^
**Alcohol use**	13 (6.3%)	12 (11.8%)	0.121^a^
**Sun exposure at work**			0.897^a^
< 1 hour/day	139 (67.5%)	70 (68.6%)	
≥ 1 hour/day	67 (32.5%)	32 (31.4%)	
**Daily sunscreen use**	59 (28.6%)	11 (10.8%)	< 0.001^a^
**Personal history of skin cancer**	13 (6.3%)	4 (3.9%)	0.442^a^
**Dermatological complaints**	129 (62.6%)	60 (58.8%)	0.536^a^

Data are presented as median (interquartile range) or number (percentage).

^a^Fisher’s exact test;

^b^Pearson chi-square test;

^c^Mann–Whitney test; RTR = renal transplant recipients; CKD = chronic kidney disease; GFR = glomerular filtration rate; CAD = coronary artery disease.

* According to The Kidney Disease Outcomes Quality Initiative of the National Kidney Foundation (KDOQI/NKF).^18^

** Calculated according to the Chronic Kidney Disease Epidemiology Collaboration group (CKD_EPI equation).^17^

Among RTR, the median time between transplant and the first dermatology visit was 99.9 months (IQR: 55.4–164.7; range: 2.0–482.0). Most of the transplant patients (45.6%) were three to 10 years post-transplant. In terms of the drug treatment, 22.3% of patients in this group were using or had already used azathioprine, 81.5% of patients were using or had used mycophenolate salts (mycophenolate sodium or mycophenolate mofetil), 88.3% of patients were using or had used calcineurin inhibitors (cyclosporine or tacrolimus), and 30.6% of patients were using or had used mammalian target of rapamycin (mTOR) inhibitors, namely sirolimus and everolimus.

### Skin disease diagnosis

Skin disorders were divided into four groups: benign, infectious, preneoplastic, and neoplastic. Non-infectious benign dermatoses were the most frequent disorders in both groups (53.9% in RTR and 60.8% in the CKD group) (Table 2). Pigmentation disorders were the most prevalent in the RTR group (11.2%), followed by adverse drug reactions (6.3%) and acne (5.8%). Among patients with CKD, the most frequent diagnoses in this category were pigmentation disorder (16.7%) and xerosis (4.9%) (Table 2). Adverse drug reactions were more prevalent in RTR than the CKD group (6.3% vs. 1.0%; P = 0.040) (Table 2).

**Table 2.** Dermatological diseases found in renal transplant recipients and chronic kidney disease patients

	RTR n = 206	CKD n = 102	P
* Benign	111 (53.9%)	62 (60.8%)	0.273^a^
Xerosis	9 (4.4%)	5 (4.9%)	0.780^a^
Pigmentation disorder	23 (11.2%)	17 (16.7%)	0.208^a^
Sebaceous hyperplasia	9 (4.4%)	0	0.032^a^
Acne	12 (5.8%)	1 (1.0%)	0.067^a^
Adverse drug reaction	13 (6.3%)	1 (1.0%)	0.040^a^
Other benign disorders	64 (31.1%)	43 (42.2%)	0.058^a^
* Infectious	81 (39.3%)	22 (21.6%)	0.002^a^
**Bacterial**			
Bacterial folliculitis	3 (1.5%)	0	0.553^a^
Impetigo	1 (0.5%)	0	1^a^
**Viral**			
Genital herpes	1 (0.5%)	0	1^a^
Herpes simplex	2 (1.0%)	0	1^a^
HPV	21 (10.2%)	2 (2.0%)	0.010^a^
Molluscum contagiosum	3 (1.5%)	0	0.553^a^
**Fungal**			
Pityriasis versicolor	17 (8.3%)	1 (1.0%)	0.009^a^
Candidiasis	8 (3.9%)	5 (4.9%)	0.765^a^
Dermatophytosis	40 (19.4%)	15 (14.7%)	0.346^a^
Systemic mycosis	1 (0.5%)	0	1^a^
**Pre-neoplastic**	21 (10.2%)	8 (7.8%)	0.679^a^
* Neoplastic	9 (4.4%)	1 (1%)	0.174^a^
Squamous cell carcinoma	3 (1.5%)	0	0.553^a^
Basal cell carcinoma	6 (2.9%)	1 (1.0%)	0.432^a^

RTR, renal transplant recipients; CKD, chronic kidney disease; HPV, human papillomavirus;

^a^Fisher’s exact test; preneoplastic (actinic keratosis);

*Because some patients had more than one type of dermatosis, the numbers for each condition may not add up to the sum for each disease subtype.

Infectious skin diseases accounted for 39.3% of the diagnoses in the RTR group and 21.6% in the CKD group (P = 0.002). The most frequent ones were, among RTR, dermatophytosis (19.4%) and HPV-related diseases (10.2%), whereas, in patients with CKD, dermatophytosis (14.7%) and candidiasis (4.9%) were prevalent. HPV-related diseases (10.2% vs. 2.0%, P = 0.01) and pityriasis versicolor (8.3% vs. 1.0%, P = 0.009) were more frequent in RTR than in patients with CKD.

The identified benign dermatological diseases are presented in terms of age subgroups in Table 3. Sebaceous hyperplasia was found only in the RTR group (in the subgroups of age: 18–39 years and 40–59 years), while adverse drug reactions were found in all RTR age subgroups, with a small number of cases and only one case in the CKD group (in the 60+ years subgroup).

**Table 3.** Diagnosis of skin disorders in renal transplant recipients and chronic kidney disease patients, stratified by age

	18–39 years old (n = 70)			40–59 years old (n = 148)			60+ years old (n = 90)		
	RTR n = 59	CKD n = 11	P-value	RTR n = 113	CKD n = 35	P-value	RTR n = 34	CKD n = 56	P-value
**Benign skin diseases**									
Xerosis	1 (1.7%)	0	1^a^	6 (5.3%)	2 (5.7%)	1^a^	2 (5.9%)	3 (5.4%)	1^a^
Pigmentation disorder	7 (11.9%)	3 (27.3%)	0.186^a^	14 (12.4%)	6 (17.1%)	0.571^a^	2 (5.9%)	8 (14.3%)	0.308^a^
Sebaceous hyperplasia	1 (1.7%)	0	1^a^	8 (7.1%)	0	0.199^a^	0	0	
Acne	8 (13.6%)	1 (9.1%)	1^a^	4 (3.5%)	0		0	0	
Adverse drug reaction	3 (5.0%)	0	1^a^	7 (6.2%)	0	0.199^a^	3 (8.8%)	1 (1.8%)	0.149^a^
Other benign disorders	15 (25.4%)	4 (36.4%)	0.474^a^	35 (31.0%)	14 (40.0%)	0.411^a^	14 (41.2%)	25 (44.6%)	0.828^a^
**Bacterial skin diseases**									
Bacterial folliculitis	1 (1.7%)	0	1^a^	2 (1.8%)	0	1^a^	0	0	
Impetigo	0	0		1 (0.9%)	0	1^a^	0	0	
**Viral skin diseases**									
Genital herpes	0	0		0	0		1 (2.9%)	0	0.378^a^
Herpes simplex	1 (1.7%)	0	1^a^	1 (0.9%)	0	1^a^	0	0	
HPV	4 (6.8%)	0	1^a^	9 (8.0%)	0	0.116^a^	8 (23.5%)	2 (3.6%)	0.005^a^
Molluscum contagiosum	2 (3.4%)	0	1^a^	1 (0.9%)	0	1^a^	0	0	
**Fungal skin diseases**									
Pityriasis versicolor	8 (13.6%)	0	0.340^a^	7 (6.2%)	1 (2.9%)	0.681^a^	2 (5.9%)	0	0.140^a^
Candidiasis	1 (1.7%)	0	1^a^	6 (5.3%)	3 (8.6%)	0.442^a^	1 (2.9%)	2 (3.6%)	1^a^
Dermatophytosis	8 (13.6%)	0	0.340^a^	21 (18.6%)	2 (5.7%)	0.106^a^	11 (32.4%)	13 (23.2%)	0.461^a^
Systemic mycosis	0	0		0	0		1	0	0.378^a^
**Pre-neoplastic and neoplastic skin diseases**									
Actinic keratosis	1 (1.7%)	0	1^a^	10 (8.8%)	1 (2.9%)	0.460^a^	10 (29.4%)	7 (12.5%)	0.057^a^
SCC	0	0		1 (0.9%)	0	1^a^	2 (5.9%)	0	0.140^a^
BCC	0	0		5 (4.4%)	1 (2.9%)	1^a^	1 (2.9%)	0	0.378^a^

^a^Fisher’s exact test; RTR, renal transplant recipient; CKD, chronic kidney disease; HPV, human papillomavirus; BCC, basal cell carcinoma; SCC, squamous cell carcinoma.

A few positive cases of two bacterial diseases (impetigo and bacterial folliculitis) were found in the study population, all in the RTR group. In the 60+ years, HPV-related diseases were more prevalent in the RTR than in the CKD group (23.5% vs. 3.6%; P = 0.005) (Table 3). Other viral diseases, such as genital herpes, herpes simplex, and molluscum contagiosum are shown in Table 3.

**Table 4.** Current and previous use of immunosuppressive drugs and dermatological diseases in renal transplant recipients (n = 206)

Immunosuppressive drugs*	NMSC			Human papillomavirus			Pityriasis versicolor		
	Present (n = 9)	Absent (n = 197)	P-value	Present (n = 21)	Absent (n = 185)	P-value	Present (n = 17)	Absent (n = 189)	P-value
**Azathioprine**	6 (66.7%)	40 (20.3%)	0.005^a^	9 (42.9%)	37 (20.0%)	0.026^a^	2 (11.8%)	44 (23.3%)	0.372^a^
**Mycophenolate salts**	5 (55.6%)	163 (82.7%)	0.062^a^	15 (71.4%)	153 (82.7%)	0.234^a^	17 (100%)	151 (79.9%)	0.047^a^
**Calcineurin inhibitors**	8 (88.9%)	174 (88.3%)	1^a^	17 (81.0%)	165 (89.2%)	0.279^a^	17 (100%)	165 (87.3%)	0.230^a^
**mTOR inhibitors**	3 (33.3%)	60 (30.5%)	1^a^	5 (23.8%)	58 (31.4%)	0.620^a^	4 (23.5%)	59 (31.2%)	0.594^a^

^a^Fisher’s exact test; NMSC = non-melanoma skin cancer; mTOR inhibitors = mammalian target of rapamycin.

* Current or previous use was considered.

Dermatophytosis was the most prevalent fungal disease, predominating in the RTR group over the CKD population, especially in the 60+ age subgroup (32.4% vs. 23.2%, respectively), but without statistical significance (Table 3). One RTR patient presented with systemic mycosis (paracoccidioid mycosis) with mucocutaneous, lymph node, and pulmonary involvement.

Actinic keratosis was more predominant in the 60+ age subgroup compared to the other age groups, with a greater prevalence in the RTR group than the CKD group (29.4% vs. 12.5%), but without statistical significance (Table 3).

**Figure 1.**
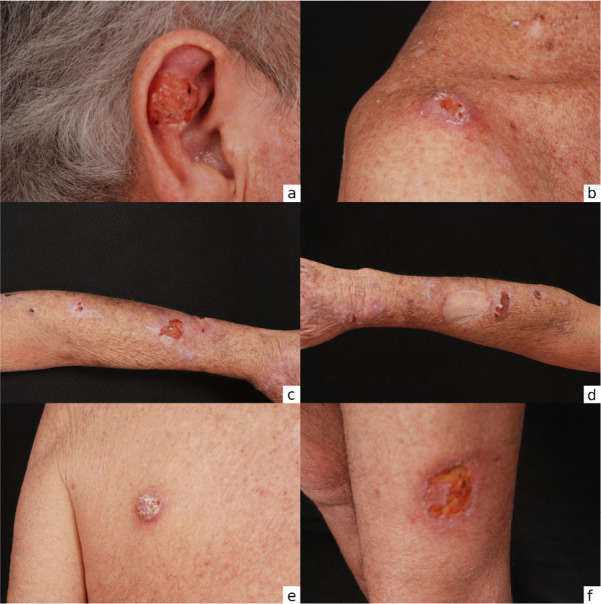
Renal transplant recipient presenting 10 basal cell carcinoma lesions at dermatology visit. Photo credit: HC/UFMG-EBSERH Dermatology Service

Neoplastic skin lesions were present in nine (4.4%) transplanted patients and only one (1.0%) subject in the CKD group (Table 2). Eighteen non-melanoma skin cancer (NMSC) lesions were found in nine patients, and one was observed in a patient with CKD (Table 2). One of these nine transplant recipients presented with ten basal cell carcinomas (BCC) at the first dermatology visit (Figure 1), while the other cases involved one lesion per patient. All nine RTR with NMSC received their transplants at least four years prior to the skin cancer, and six (66.6%) of these patients already had a history of skin cancer.

Squamous cell carcinoma (SCC) was found in 5.9% of individuals in the 60+ age subgroup in the RTR group and not found in the CKD group in this age range; the frequency of BCC was 4.4% in the RTR group compared to 2.9% in the CKD group among patients in the age range of 40–59 years (Table 3).

In terms of immunosuppressive drugs, of the 21 RTR patients with HPV, nine (42.9%) patients were using or had used azathioprine (P = 0.026) (Table 4). Of the 17 RTR who presented with pityriasis versicolor, 17 (100%) patients were using or had previously used mycophenolate salts (P = 0.047). Of the nine NMSC patients, six (66.7%) patients were using or had used azathioprine (P = 0.005) (Table 4).

Among patients with CKD, no statistically significant association was found between the CKD stage and diagnosed skin diseases.

## DISCUSSION

This study investigated patients with CKD and RTR: two populations with a similar distribution of skin phototypes, but distinct demographic profiles and kidney disorders. Patients with CKD were older, had a higher frequency of comorbidities and smoking, and had lower glomerular filtration rates than RTR. In contrast, RTR had a higher frequency of regular sunscreen use than patients with CKD. It is likely that nephrologists may be less persuasive about sun protection measures in patients with CKD than in individuals who have received organ transplants. However, daily sunscreen use is insufficient for adequate protection, and reapplication is necessary every three hours.^30^ Additional physical protection measures, such as ultraviolet (UV)-protective clothing, hats, sunglasses, and shade, are simple and effective ways to protect individuals from UV radiation and preventing NMSC.^31^

The frequency of skin infections observed in our population was lower than that in other studies.^15,34,35^ Skin infections predominated within the first 3–4 years after transplantation,^28^ and more than 3 years had elapsed after transplantation in 84.9% of our RTR sample. There is evidence that increased susceptibility to bacterial, fungal, and viral cutaneous infections in patients with CKD varies between 28 and 70%.^7^ Patients with CKD have impaired cellular immunity due to a decreased T lymphocyte cell count, which could explain the high prevalence of infection in those patients.^36^ However, the literature on cutaneous infections in individuals with CKD is sparse.

HPV-related skin disease was the most common viral infection, and its frequency was higher in RTR than the CKD group (10.2% vs. 2.0%), in line with previous studies, as a result of chronic immunosuppression.^29,37^ Previous research found that 15% of patients present with cutaneous viral warts during the first year after renal transplant, and that this rate reached 92% after a period of 15 years.^38^ In approximately 60% of our sample, ten years had not passed since transplantation. In elderly patients, the difference in HPV among RTR and patients with CKD was remarkable (23.5% vs. 3.6%, P = 0.005), which can be partially explained by the persistence of HPV in old age.^37^ Furthermore, the age subgroup of 60+ years had proportionally more individuals (50%) at least 120 months post-transplantation, implying a longer period of immunosuppression and increased time of HPV persistence. Despite being a viral skin disease of concern owing to its oncogenic potential to increase the risk of SCC in immunosuppressed patients,^12,29^ as it facilitates the accumulation of DNA mutations induced by UV radiation,^39^ none of the three patients with SCC in our study had HPV-related disease.

Renal transplant recipients also have a higher frequency of infectious skin diseases, pityriasis versicolor, sebaceous hyperplasia, and adverse drug reactions than patients with CKD. In terms of bacterial skin diseases, only impetigo and folliculitis were diagnosed in the RTR, probably because of the low number of participants in our sample. Moreover, bacterial infections are more prevalent during the first years after transplantation (only 15% of our sample was less than three years post-transplantation), and acute and benign diseases are often treated by an assistant physician without a referral to a dermatologist. No bacterial skin disease was diagnosed in the CKD group.

In terms of immunosuppressant use, NMSC and HPV infections were more frequent in RTR who received azathioprine. Pityriasis versicolor was observed to be associated with the use of mycophenolate salts in RTR. No association was found between the CKD stage and skin disorders. Immunosuppressive agents play important roles in the development of dermatological diseases. With regard to skin cancer in RTR, these drugs have direct carcinogenic action and reduce immunological surveillance.^29^ Azathioprine and cyclosporine may directly or indirectly interact with UV radiation to enhance its carcinogenic effects.^29^ A higher prevalence of azathioprine use was observed in RTR with NMSC compared to RTR without skin cancer (P = 0.005). Other immunosuppressants were not associated with skin cancer in the present study.

As for immunosuppressive agents and infectious dermatoses, of the 21 RTR with HPV, 42.9% used azathioprine; among the RTR without a diagnosis of HPV, only 20% used the same drug (P = 0.026). This is corroborated by a Brazilian study in which patients who used this drug had a higher incidence of viral warts.^23^ The use of mycophenolate salts was more frequent in patients with pityriasis versicolor than in those who were not treated with this medication (P = 0.047). In the RTR group, 81.5% of patients were using or had already used mycophenolate salts. It was, therefore, by chance that 100% of patients who presented with pityriasis versicolor had used this drug.

BCC was more prevalent than SCC in the RTRs. In the general population, BCC predominates over SCC at a ratio of 4:1.^19^ However, this ratio is reversed in solid organ transplants, and becomes more pronounced as more time elapses post-transplantation.^19,20^ We found a higher proportion of BCC compared to SCC (2:1) in the RTR group; these findings are consistent with observations by Lima et al.^21^ Another Brazilian study and a research on organ transplant recipients in the Mediterranean has also reported similar ratios.^22^ The genetic background, higher phototypes, and phenotypic characteristics could be responsible for this phenomenon; however, another Brazilian study by Hayashida et al.^23^ found a BCC:SCC ratio of 1:2.4 with a minimum follow-up of three years.^23^

Notably, over the past five years, some follow-up studies have found lower BCC:SCC ratios.^20,24,25^ These results can be partially explained by the reduced trend of SCC incidence over the past 20 years in solid organ transplant recipients.^26,27^ This decline is likely caused by less aggressive and more individualized immunosuppression therapy.^26^

One of the most important extrinsic factors related to the increased incidence of NMSC is exposure to UV radiation.^19,29^ Of the RTR patients with NMSC, 66.6% of patients reported no exposure or up to one hour of sun exposure per day at work. There is evidence that in temperate climates, 35–50% of organ transplant recipients will develop one or more skin cancers by the tenth year after transplantation; this number may increase to more than 80% in countries with higher rates of UV radiation.^30^ Regular sun protection is of utmost importance for immunosuppressed patients.

Patients with CKD receiving conservative treatment have demonstrated a higher incidence of kidney and urinary tract cancers than the general population;^32,33^ however, the incidence of NMSC is unclear.^32^ Wang et al.^32^ found that predialysis patients (stage 5 CKD) have a greater risk of developing NMSC than the general population, with a standardized incidence ratio (SIR) of 1.14. In our study, only one patient with CKD had BCC.

The most common fungal infections occurring in RTR are superficial mycoses.^28^ Dermatophytosis was the most common mycosis found; however, no difference was observed between RTR and patients with CKD or age subgroups. The prevalence of superficial mycoses in RTR varies in the literature (16–60%), probably in accordance with the study type, length of follow-up, and geographic region.^28,34^ Charu^7^ found a prevalence of 16.9% and Thomas^36^ found a prevalence of 1.01% in patients with CKD.

Among benign diseases, sebaceous hyperplasia was more frequent in RTR than in individuals with CKD. Sebaceous hyperplasia was found only in the RTR group, particularly in the age subgroup of 40–59 years. It is observed as a complication in 30% of patients using cyclosporine,^28^ as this drug may be partly eliminated through the sebaceous glands, leading to frequent pilosebaceous lesions.^11^

A few cases of xerosis were observed in either group, with no significant differences. Our findings were lower than the prevalence rates observed by other authors, 50–80% in CKD subjects.^7,8^ The low prevalence observed in the CKD group may be partly explained by the fact that most of our patients with CKD (77.4%) were stage 3B or less; dermatoses, including xerosis, are more prevalent in the later stages of CKD.^7,8^

This study had some limitations. Acute dermatoses may have been underestimated owing to the study design, as skin lesions may not have been present on the day of the dermatologist’s consultation. The data included in this study refer only to the first consultation, which makes it difficult to accurately characterize the spectrum of diseases presented over time. Finally, this study was based on a single-center analysis, which limits the generalizability of the results. However, this method has several strengths. Despite being a single-center study, this is a reference center for transplants in the state of Minas Gerais, and all patients underwent detailed assessment by a team of dermatologists with extensive expertise. Additionally, the study assessed patients from a highly miscigenic population in a tropical region.

## CONCLUSIONS

This study of patients monitored at a reference center for nephrology and renal transplantation found more skin infections in kidney transplant recipients than in patients with CKD. A multidisciplinary team, including dermatologists, must know how to diagnose, treat, and implement skin disease prevention measures in these populations. Therefore, the skin of these patients should be routinely evaluated to manage dermatological diseases, especially neoplasms.
